# Comparative Assessment of Crestal Bone Levels in Immediate Versus Delayed Implant Placement With and Without Bone Graft

**DOI:** 10.7759/cureus.58418

**Published:** 2024-04-16

**Authors:** Vijay Mishra, Ashutosh Dixhit, Prerna Kataria, Rajesh K Thakur, Awanindra K Jha, Bipin K Yadav

**Affiliations:** 1 Oral and Maxillofacial Surgery, Uttar Pradesh University of Medical Sciences, Etawah, IND; 2 Dentistry, All India Institute of Medical Science, Rishikesh, Rishikesh, IND; 3 Periodontology, DJ College of Dental Sciences and Research, Modinagar, IND; 4 Periodontology, Faculty of Dentistry, Uttar Pradesh University of Medical Sciences, Etawah, IND; 5 Orthodontics, Rajendra Institute of Medical Sciences, Ranchi, IND

**Keywords:** prosthesis, immediate implant placement, delayed implant placement, dental implant, bone graft

## Abstract

Background

Presently, modern regenerative and surgical techniques for immediate implant placement in prepared sockets with soft tissue grafts and bone substitutes have helped eliminate concerns about bone deficiency. This also allowed the placement of dental implants based on prosthodontic needs.

Aim

The present study aimed to comparatively assess dental implant healing following immediate implant placement with or without bone graft and dental implant healing after delayed implant placement with or without bone graft.

Methods

The study included 120 study subjects that were divided into two groups. Group I for immediate implant placement with or without bone graft (n=60) and Group II for delayed implant placement with or without bone graft (n=60). These two groups were further divided into subgroups. Group I subjects were further divided into two subgroups, where Group A (n=30) subjects underwent immediate implant placement with bone graft and Group B (n=30) subjects were given immediate implant placement without bone graft. Group II participants were further divided into two subgroups, where Group C (n=30) subjects underwent delayed implant placement with bone graft and Group D (n=30) subjects underwent delayed implant placement without bone graft. In the two groups, crestal bone levels were compared radiographically preoperatively and postoperatively at the immediate postoperative time, three months, and six months.

Results

More reduction in the crestal bone level was seen in the immediate implant placement group at three and six months postoperatively compared to the delayed implant placement group. A non-significant reduction in crestal bone levels was seen in the immediate implant placement with bone graft group with p>0.05 at three and six months compared to immediate implant placement without bone graft. Similar, non-significant crestal bone loss was seen in the delayed implant placement with bone graft group at three and six months compared to delayed implant placement with bone graft.

Conclusions

This study concluded that healing of crestal bone in the delayed implant placement group with or without bone graft is better in comparison to the immediate implant placement group with or without bone graft.

## Introduction

With the improved life expectancy, teeth retention is needed for a longer duration, warranting their replacement in cases of missing teeth. With the advances in various technologies and techniques in the field of dentistry, the introduction of osseointegrated dental implants presents a turning point for missing tooth rehabilitation. The use of endosseous dental implants is increasingly becoming popular with global clinical applications concerning the prosthetic standard of treatment owing to their high success predictability and different therapeutic options [1]. 

Osseointegration is a vital factor that governs the success and outcome of dental implants. Other factors that are considered vital for osseointegration in dental implants are accessible bone condition, implant geometry, and placement time, whether delayed or immediate [2]. Considering the increase in demand for shorter treatment durations in dental implant treatment, various loading protocols and surgical positioning have been formulated and used. To consider the implant placement time, various strategies that are used include traditional delayed placement, early placement, and immediate placement [3].

Delayed implant placement describes the conventional technique of placing dental implants that has the benefit of having less microbial infection following the placement of dental implants. Delayed implants are placed after 12 weeks or more of extraction; early implants are placed less than 12 weeks after extraction but not immediately following extraction; and immediate dental implants are placed into fresh extraction sockets. Delayed implant placement is a vital technique for placing dental implants, as the environment is highly conducive to healing and developing the appropriate osseointegration. Fresh extraction sockets are filled with blood and various products from the tooth as dental plaque, whereas these risks are eliminated in delayed implants, making them less susceptible to microbial infections [4].

After tooth extraction, the recovery period is traditionally associated with the insertion of a dental implant. Earlier dental implants were conventionally placed after 12 weeks of extraction, which was considered adequate for the healing of the extraction socket. Various studies are questioning the effectiveness of this healing time. Recently, the conventional treatment time has been reduced with a reduction in time between implant placement and loading [5].

In this study, bone healing was assessed by measuring the crestal bone level. Both processes are interrelated and coordinated. Eliminating the term healing from the article and using only the crestal bone level assessment. As the majority of the subjects need low treatment costs with less invasive and simple surgical procedures, it is desired to evaluate the need for various techniques of bone-reconstructive therapy to attain immediate implant osseointegration. Hence, the size and form of the peri-implant bone defects that heal spontaneously with the bone must be made [6]. 

The objective of the study was to comparatively assess the changes in the crestal bone levels following delayed and immediate dental implant placement. The hypothesis for the study was that crestal bone levels are higher in immediate implant placement compared to delayed implant placement. The present study aimed to comparatively assess the crestal bone alterations and bone healing after immediate and delayed implant placement with or without the bone grafts.

## Materials and methods

This comparative clinical study was done after clearance was given by the Institutional Ethical Committee at Uttar Pradesh University of Medical Sciences, Etawah, Uttar Pradesh, India (IRB: 1-20/2022). The study subjects were from the Department of Oral and Maxillofacial Surgery of the Institute. Before the study, all subjects gave written and verbal informed consent. The study was conducted over a period of 12 months, from January 2022 to December 2022.

The study included subjects that needed dental implants in either delayed implant placement or immediate implant placement following extraction in either completely edentulous or partially edentulous regions in either the maxilla or mandible. Before inclusion, we conducted an initial comprehensive assessment of the subjects. The inclusion criteria for the study were subjects in the age range of 18-65 years, subjects with no deformity or illness that can affect the implant osseointegration, subjects with sufficient bone quality and quantity as assessed clinically and radiographically, subjects conscious of their hygiene who gave informed consent for participation, subjects with complete tooth eruptions, and subjects with complete facial growth. The exclusions were subjects with local or systemic conditions that rendered them not eligible for study participation.

The study included 120 subjects from both genders and in the age range of 18-65 years who needed aesthetic and functional rehabilitation secondary to unrestorable or decayed teeth and had a partially edentulous arch, maxilla, or mandible. The sample size was determined by the availability of patients at the institute. The included subjects that needed dental implant placement either immediately following extraction or delayed placement of the dental implant in either a partial or completely edentulous maxillary or mandibular arch. These were randomly divided into one of the two groups, where Groups I and II had 60 subjects each, with subgroups A and B having 30 subjects each that underwent immediate implant placement. Osstem Dental Implant (TSIII ®) provided the dental implants. The remarkable dual thread, self-tapping capability, and effective corkscrew thread of the TS implants set them apart from the competition. They also provide long-term primary stability. They have a morse taper connection, providing a superior connection between the implant and the abutment. There are no microgaps that minimize bone loss through microbial restriction.

These participants from Group I were subsequently split into two subgroups, with Group A receiving immediate implant insertion along with a bone graft. To preserve the alveolar ridge or socket through osteoconduction, a hydroxyapatite bone graft, which has the best bone regeneration qualities, was utilized. This also has good hardness and a high bone acceptance rate. Group B subjects were given immediate implant placement without a bone graft. Group II comprised subjects who underwent delayed implant placement. These Group II participants were further divided into two subgroups, where Group C subjects underwent delayed implant placement with bone graft and Group D subjects underwent delayed implant placement without bone graft. 

Before placement of the dental implants, a comprehensive clinical examination was done on all the subjects, along with a radiographic assessment using intra-oral periapical radiographs or an orthopantomogram. The study assessed the bone levels with intraoral periapical radiographs to allow the application of study results in the same place and in subjects with limited resources. Crestal bone levels were compared radiographically in the two groups, and healing and crestal bone levels were compared in two groups preoperatively and postoperatively at the immediate postoperative time, three months, and six months.

We statistically analyzed the collected data using IBM SPSS Statistics for Windows, Version 21 (Released 2012; IBM Corp., Armonk, New York, United States) to perform a t-test and a one-way analysis of variance (ANOVA) test. The normal distribution of data was tested analytically. The data were expressed as mean and standard deviation, frequency, and percentage. Statistical significance was kept at a p-value of <0.05. To evaluate the change in parameters of any group before and after surgery, repeated measurements and ANOVA were used for a simple t-test.

## Results

The study included 120 subjects from both genders and in the age range of 18-65 years who needed esthetic and functional rehabilitation secondary to unrestorable or decayed teeth and had a partially edentulous arch, maxilla, or mandible. The demographic data of study participants is summarized in Table 1.

**Table 1:** Demographic data of study participants n: number of participants

Characteristics	Group I	Group II
Mean age (years)	38.6±8.32	37.8±6.66
Age range (years)	18-65	18-65
Gender n (%)		
Males	38 (63.3)	33 (55)
Females	22 (36.6)	27 (45)
Edentulous site n (%)		
Maxilla	36 (60)	35 (58.3)
Mandible	24 (40)	25 (41.6)

When the crestal bone levels of the two study groups were examined, it was found that the immediate implant placement group had considerably greater preoperative crestal bone levels than the delayed implant placement group (p<0.001), as determined by radiographic analysis using built-in digital indicators.

Immediately after implant placement, crestal bone level in Group I was 0.017±0.007 which was significantly higher compared to Group II, where it was 0.013±0.002 mm, depicting a statistically significant difference with p<0.001. A similar statistical difference was seen at three months and six months postoperatively with higher crestal bone levels in the immediate implant placement group with p<0.0001 as shown in Table 2.

**Table 2:** Comparison of crestal bone levels (in mm) in Group I (immediate implant placement) and Group II (delayed implant placement) study subjects using one-way ANOVA SD: standard deviation; ANOVA: analysis of variance

Assessment time	Groups	Crestal bone level (Mean ± S. D)	p-value
Preoperative	I	0.016±0.007	<0.0001
	II	0.012±0.002	
Immediately after placement	I	0.017±0.007	<0.0001
	II	0.013±0.002	
3 months	I	0.017±0.007	<0.0001
	II	0.012±0.003	
6 months	I	0.016±0.007	<0.0001
	II	0.007±0.006	

On comparing the crestal bone levels following immediate implant placement with or without bone grafts, it was seen that preoperatively, crestal bone levels (the significance level mentioned below) were comparable preoperatively in immediate implant placement sites with or without bone grafts (p=0.315). Immediately following implant placement, crestal bone levels were 0.018±0.004 and 0.016±0.003 mm at sites with and without bone graft use, respectively. The difference was statistically non-significant, with p=0.271. A similar non-significant difference was seen at three and six months postoperatively for crestal bone levels at immediate implant placement sites with or without bone graft, with p=0.427 and p=0.750, respectively, as depicted in Table 3. 

**Table 3:** Comparison of crestal bone levels (in mm) in two subgroups A (immediate implant placement with bone graft) and B (immediate implant placement without bone graft) of Group I (immediate implant placement) with or without bone graft using one-way ANOVA SD: standard deviation; ANOVA: analysis of variance

Assessment time	Groups	Crestal bone level (Mean ± S. D)	p-value
Preoperative	A	0.017±0.004	0.315
	B	0.015±0.003	
Immediately after placement	A	0.018±0.004	0.271
	B	0.016±0.003	
3 months	A	0.019±0.003	0.427
	B	0.018±0.02	
6 months	A	0.017±0.003	0.750
	B	0.016±0.004	

For comparison of crestal bone levels following delayed implant placement with or without bone graft, a non-significant statistical difference was seen in delayed implant placement sites with or without bone graft with a non-significant higher bone level in delayed implant placement sites without the bone graft with p=0.315, 0.271, 0.427, and 0.750, respectively, at preoperative, immediately following implant placement, three months after implant placement, and six months after implant placement, as summarized in Table 4. 

**Table 4:** Comparison of crestal bone levels (in mm) in two subgroups C (delayed implant placement with bone graft) and D (delayed implant placement without bone graft) of Group II (delayed implant placement) using one-way ANOVA SD: standard deviation; ANOVA: analysis of variance

Assessment time	Groups	Crestal bone level (Mean ± S. D)	p-value
Preoperative	C	0.013±0.002	0.315
	D	0.010±0.001	
Immediately after placement	C	0.014±0.002	0.271
	D	0.012±0.002	
3 months	C	0.013±0.003	0.427
	D	0.011±0.03	
6 months	C	0.007±0.002	0.750
	D	0.007±0.002	

Concerning the comparison of crestal bone levels following immediate and delayed implant placement with bone graft, significantly higher crestal bone levels were seen at immediate implant placement sites with bone grafts with 0.17±0.004, 0.018±0.004, 0.019±0.003, and 0.017±0.003 mm respectively at preoperative, immediate postoperative, three months, and six months following immediate implant placement with bone graft compared to 0.013±0.002, 0.014±0.002, 0.013±0.003, and 0.6007±0.002 mm following delayed implant placement with bone graft and respective p-values of 0.004, 0.003, 0.001, and 0.001 (Table 5).

**Table 5:** Comparison of crestal bone levels (in mm) in subgroups A (immediate implant placement with bone graft) and C (delayed implant placement with bone graft) using one-way ANOVA SD: standard deviation; ANOVA: analysis of variance

Assessment time	Groups	Crestal bone level (Mean ± S. D)	p-value
Preoperative	A	0.17±0.004	0.004
	C	0.013±0.002	
Immediately after placement	A	0.018±0.004	0.003
	C	0.014±0.002	
3 months	A	0.019±0.003	0.001
	C	0.013±0.003	
6 months	A	0.017±0.003	0.001
	C	0.007±0.002	

The study results showed that on comparing the crestal bone levels following immediate and delayed implant placement without bone graft, significantly higher crestal bone levels were seen for immediate implant placement without bone graft groups with p=0.04, 0.003, 0.001, and 0.001, respectively, at preoperative, immediately following implant placement, three months, and six months postoperatively, as shown in Table 6.

**Table 6:** Comparison of crestal bone levels (in mm) in subgroups B (immediate implant placement without bone graft) and D (delayed implant placement without bone graft) using one-way ANOVA SD: standard deviation; ANOVA: analysis of variance

Assessment time	Groups	Crestal bone level (Mean ± S. D)	p-value
Preoperative	B	0.015±0.003	0.004
	D	0.010±0.001	
Immediately after placement	B	0.016±0.003	0.003
	D	0.012±0.002	
3 months	B	0.018±0.02	0.001
	D	0.011±0.03	
6 months	B	0.016±0.004	0.001
	D	0.007±0.002	

## Discussion

The present study included 120 subjects from both genders and in the age range of 18-65 years who needed esthetic and functional rehabilitation secondary to unrestorable or decayed teeth and had a partially edentulous arch, maxilla, or mandible. On comparing the crestal bone in two groups of study subjects, preoperatively, crestal bone levels were significantly higher in the immediate implant placement group compared to delayed implant placement (p<0.001). Immediately after implant placement, crestal bone level in Group I was 0.017±0.007, which was significantly higher compared to Group II, where it was 0.013±0.002 mm, depicting a statistically significant difference with p<0.001. A similar statistical difference was seen at three months and six months postoperatively, with higher crestal bone levels in the immediate implant placement group (p<0.0001). These results were consistent with the studies of Schropp L et al. [7] in 2003, where authors reported statistically significant differences in crestal bone level and healing after delayed and immediate dental implant placement, as seen in the present study, and with Vandeweghe S et al. [8] in 2011, where authors reported a mean bone loss of 0.38 mm on periapical radiographs after implant placement. This was attributed to the normal physiological bone remodeling of the alveolar bone by the authors.

On comparing the crestal bone levels following immediate implant placement with or without bone grafts, it was seen that preoperatively, crestal bone levels were comparable at immediate implant placement sites with or without bone grafts (p=0.315). Immediately following implant placement, crestal bone levels were 0.018±0.004 and 0.016±0.003 mm at sites with and without bone graft use, respectively. The difference was statistically non-significant, with a p-value of 0.271. A similar non-significant difference was seen at three and six months postoperatively for crestal bone levels at immediate implant placement sites with or without bone graft, with p=0.427 and 0.750, respectively. These results were in agreement with Diago MA et al. [9] in 2011 and Paolantonio M et al. [10] in 2001, where authors reported that no significant difference was seen following immediate dental implant placement with or without bone graft at all the assessment times, as seen in the results of the present study. These results can be attributed to the fact that less bone loss is seen following immediate implant placement, as bone preservation is attained by the dental implants placed immediately, posing non-significant results after bone graft use.

The study results showed that for comparison of crestal bone levels following delayed implant placement with or without bone graft, a non-significant statistical difference was seen in delayed implant placement sites with or without bone graft with a non-significant higher bone level in delayed implant placement sites without the bone graft with p=0.315, 0.271, 0.427, and 0.750, respectively, at preoperative, immediately following implant placement, three months after implant placement, and six months after implant placement. These results were in line with Chen ST et al. [11] in 2004 and Schwartz-Arad D et al. [12] in 1997, where authors suggested no significant difference following delayed dental implant placement with or without bone graft at all the assessment times, as seen in the results of the present study. In delayed implant placement, bone graft showed no significant difference concerning crestal bone loss, as the bone remodeling and loss were already there before placing the dental implants, and osseointegration stabilized the implant after placement.

For the comparison of crestal bone levels following immediate and delayed implant placement with bone graft, significantly higher crestal bone levels were seen at immediate implant placement sites with bone grafts with 0.17±0.004, 0.018±0.004, 0.019±0.003, and 0.017±0.003 mm respectively at preoperative, immediate postoperative, three months, and six months following immediate implant placement with bone graft compared to 0.013±0.002, 0.014±0.002, 0.013±0.003, and 0.6007±0.002 mm following delayed implant placement with bone graft and respective p-values of 0.004, 0.003, 0.001, and 0.001. These findings were similar to the studies of Nemcovsky CE et al. [13] in 2000 and Clementini M et al. [14] in 2013, where authors reported significantly higher crestal bone levels in immediate implant placement cases compared to delayed implant placement. The avoidance of bone loss resulting from remodeling after extraction is the reason for the higher bone levels in cases of immediate implant insertion.

Additionally, it was observed that when crestal bone levels were compared between immediate and delayed implant placement groups without bone graft, the immediate implant placement group without bone graft group showed significantly higher crestal bone levels at preoperative, immediately following implant placement, three months, and six months postoperatively (p=0.04, 0.003, 0.001, and 0.001, respectively).

These results were comparable to Block MS et al. [15] in 2009 and Viswambaran M et al. [16] in 2014, where authors reported significantly higher crestal bone levels, as seen in the present study without the use of bone substitutes. The higher bone levels in immediate implant placement cases can be attributed to the prevention of bone loss following extraction. 

Limitations

The study had a few drawbacks, including a limited study sample size, a shorter follow-up period, and an inability to examine the long-term consequences of dental implant installation. Since every participant came from a comparable region, it was not possible to apply the study's findings broadly. The study did not take the study participants' eating habits into account, which could have a negative impact on the crestal bone levels and the stress on the dental implants. Dental implants' load and vertical force transmission may be impacted by implant design and length, which was not taken into account.

## Conclusions

After accounting for its limitations, the current study concludes that, compared to the immediate implant placement group, the delayed implant placement group, with or without bone graft, had improved radiographic and clinical bone repair. To further elucidate the matter, additional longitudinal studies with extended follow-up periods and a greater number of patients with varying implant designs are required.
